# Novel sulfonated poly (vinyl alcohol)/carboxy methyl cellulose/acrylamide-based hybrid polyelectrolyte membranes

**DOI:** 10.1038/s41598-022-26489-0

**Published:** 2022-12-20

**Authors:** Atia Mahmoud, Alaa Fahmy, Abdelrahman Naser, Mohamed Abu Saied

**Affiliations:** 1 Chemistry Department, Faculty of Science, Al-Azhar University, Cairo, 11884 Egypt; 2 Polymeric Materials Research Department, Advanced Technology and New Materials Research Institute (ATNMRI), The City of Scientific Research and Technological Applications (SRTA-City), New Borg Al-Arab City, 21934 Alexandria Egypt

**Keywords:** Chemistry, Energy science and technology, Materials science, Nanoscience and technology

## Abstract

Novel polyelectrolytic hybrid membranes are prepared by blending carboxy methyl cellulose (CMC)-polyvinyl alcohol (PVA)-acrylamide (AA). Succinic acid and chlorosulfonic acid (CSA) are employed as crosslinkers and modifiers, respectively. Additionally, carboxylated carbon nanotube (CCNT) and sulfonated activated carbon (SAC) as fillers are used to attain appropriate chemical and mechanical stability for use as polyelectrolyte membranes (PEM). CMC, PVA, and AA are mixed and treated with CSA, CCNT, and SAC in different concentrations. First, CMC/PVA/AA solution is modified using CSA to produce a sulfonated polymeric matrix. Second, a different amount of CCNT or SAC was added as a filler to enhance the ion exchange capacity (IEC), ionic conductivity, and chemical stability. Third, the solution is cast as polyelectrolytic membranes. Chemical interactions between CMC, PVA, AA and other membrane components were confirmed using various characterization techniques such as Raman scattering spectroscopy and Fourier Transform Infrared (FTIR). Furthermore, mechanical strength, methanol uptake, gel fraction, ion exchange capacity (IEC), proton conductivity (PC), chemical and thermal stability were determined as functions of varied membrane modification components. Results reveal that the increase of CSA, CCNT and SAC is leading to increase the IEC values reaching 1.54 mmol/g for (CMC/PVA-4% CSA), 1.74 mmol/g for (CMC/PVA-4%CSA-2%CCNT) and 2.31 mmol/g for (CMC/PVA-4% CSA-2% SAC) comparing to 0.11 mmol/g for non-modified CMC/PVA/AA membrane. Sequentially, the proton conductivity value is changed from 1 × 10^–3^ S/cm in non-modified CMC/PVA/AA membrane to 0.082 S/cm for (CMC/PVA-4% CSA), 0.0984 S/cm for (CMC/PVA-4%CSA-2%CCNT) and 0.1050 S/cm for (CMC/PVA-4% CSA-2% SAC). Such results enhance the potential feasibility of modified CMC/PVA/AA hybrid as polyelectrolytic membranes.

## Introduction

Currently, direct methanol fuel cells (DMFC) represent an inventive alternative for current power sources among the other fuel cell (FCs) types. DMFC had significant importance in the recent few years because of its low operating temperature, high-power density, high energy conversion efficiency and become less expensive fuel^1^. Rather, DMFC is considered one of the important tools that take a part in turning to carbon–neutral operations^2^.

The electrolytic membrane is the operating heart of the cell, it is responsible for conducting protons (H^+^) from the anode part to the cathode. Some of the important characteristics of the polyelectrolytic membrane are good conductors for protons (H^+^), electrically insulators, very low methanol cross-over chemically stable, and withstand during cell operating conditions^3^.

However, a major restriction of DMFC is the dropout of methanol through the electrolytic membrane and the production of highly reactive polymeric electrolytes with optimum water management. In addition, fouling is one of the problems that may be affected the polymeric membranes^4^ by decreasing their efficiency by blocking the proton-binding sites. Particularly, the fouling that happened by fuel impurities or other biological and chemical interactions^5^. The fouled polymeric membrane must be subject to recovery process or replaced, this would increase the cost-operating efficiency. Rana et al.^6^ found that, the addition of silver salts to polymeric membrane leading to an improvement of anti-fouling effect.

Nowadays Nafion^®^ is widely used as an electrolyte in polymer electrolyte membrane fuel cell (PEMFC) but is limited by methanol crossover and water management problems^7,8^.

The polyelectrolytic membrane can be produced using different types of polymers after the modification process to enhance the desired character and improves their ionic conductivity. Modification can be done through blending and/or chemical treatment^9^. Introducing inorganic materials such as HAP^10^, CNT^11^ or GO^12^ with polyelectrolytic membranes leads to improving protonic conductivity and mechanical stability in addition to enhancing barrier effect which is the important property of preventing fuel cross-over^13^.

For more effectiveness, functionalization of the inorganic blend is the key factor to producing a filler with high surface active (interfacial area), good compatibility and prevented sedimentation or agglomeration during the polyelectrolytic membrane production process^14^. CNTs are one of the most attractive materials as inorganic membrane fillers owing to their high aspect ratio and surface area with low density. It has a limited dispersion in the polymeric membrane owing to its low functional groups. The polymeric blend can be improved by the addition of CNTs or modified CNTs through the functionalization of CNTs with –COOH, –OH, or –SO_3_H groups^15,16^. Not only mechanical or thermal stability improved but also ionic conductivity growing as a result of micro-channels provided by polymer blend around CNTs. Rambabu et al.^17^ discussed modifying CNTs by introducing –SO_3_H groups using polystyrene sulfuric acid to prepare the SPEEK nanocomposite membrane. The protonic conductivity and mechanical stability were improved particularly with increasing sulfonation process rather than overall performance^13^.

Another attractive filler is the activated carbon (AC) molecular sheet. The advantage of AC is attributed to its excellent thermal^12^, mechanical^18^ and transport properties. However, AC is difficult to exfoliate in a polymer matrix due to its high cohesive force between them. Thus, AC is functionalized with other groups such as (–SO_3_H) to impart specific features and interactions^19^.

Rahul et al.^20^ stated that, the salt incorporation in polymeric membrane produces relatively higher proton conductivity (~ 10^–5^ to ~ 10^–3^ S/cm), flexible geometry and electrochemical stability. The benefit of protonic conducting components is their advanced dynamics for the proton association. The protonic conducting blends as (NH_4_^+^) are good proton conducting sources in the development of some types of polymeric membranes^21^. Leena et al.^22^ provided that NH_4_SCN can increase the (H^+^) transferred across cell parts through the polymeric membrane. several studies explained that^23^ the transportation based on polymeric ammonium complexes system is mainly due to NH_3_–H^+^.

PVA-ZnO-based polyelectrolyte DMFC membrane was prepared by Diyan Ul Imaan^24^ through impregnating zinc oxide nanoparticles in a cross-linked PVA matrix. The obtained membrane possesses high water uptake (105%), IEC (0.78 meq/g) and low ionic conductivity (3.9 mS/cm). In the same way, Tripathi and Shahi^16^ prepared N-p-carboxy benzyl chitosan-silica-PVA proton exchange membrane for DMFC by sol–gel method. The obtained membrane exhibits low ionic conductivity as a result of weak proton conducting carboxylic groups. The conductivity and stability improved through the introduction of high charge density (–SO_3_H) groups. Asnag et al*.*^25^ successfully prepared PVA-CMC based nanocomposite film via a solution casting technique for electrical and optical properties. Polyacrylamide/Nafion electrolyte membrane^7^ shows an increase in water and methanol uptake with raising the amount of Polyacrylamide in the membrane. In contrast, the IEC shows decreasing by inserting Polyacrylamide into Nafion^®^.

Therefore, in this work, the research was conducted to find an alternative membrane for DMFC based on sulfonated CMC and PVA. Due to the non-toxicity, cheapness, biodegradability, and good matrix forming of CMC and PVA, they are selected for this study. Acrylamide (AA) blend is added to improve the ionic conductivity and facilitate proton transportation across the membrane through the formation of positively charged ammonium (NH_4_^+^) ions^7,26^. The blending of CMC with PVA and AA followed by sulfonation using chlorosulfonic acid (CSA) with the addition of carboxylated carbon nanotube (CCNT) or sulfonated activated carbon (SAC) as fillers to improve the blocking methanol crossover and enhance the IEC and proton transportation property of the membrane with enhanced electrochemical performance.

Modification of CMC/PVA/AA based membranes through introducing of CCNT and SAC are providing reinforcement and will give a winding path for methanol cross-over through the membrane in addition maintain water swell-ability which may be overcome the methanol-crossover and water management fuel cell problems^27^. Furthermore, the introducing of CCNT and SAC can improve the mechanical and thermal stability in addition to its involvement to enhance ionic conductivity by providing a large interfacial area and ionic channels.

## Materials and methods

### Materials

CMC (average Mw = 90,000 g/mol, DS = 0.7) was obtained from Acros Organics, Fisher Scientific, UK. PVA (typically average Mw = 124,000 g/mol, 95–96.5% hydrolyzed) was obtained from Fisher Scientific, UK. Chlorosulfonic acid (M = 116.52 g/mol, purity 99%, density 1.753 g/mL at 25 °C) was purchased from Merck, Germany. Multi-wall CCNT (density 2.1 g/cm^3^, diameter 10–20 nm, length 10–30 nm, –COOH content 2 wt%) was achieved from XFNANO, Jiangsu, China. Activated carbon (Mw = 12.01 g/mol, sulfur content 0.15%, acid solubility 2.0%) bought from Uni-CHEM chemical reagents, Korea.

### Preparation of CMC/PVA/AA based hybrid polyelectrolytic membranes

**Scheme 1:**
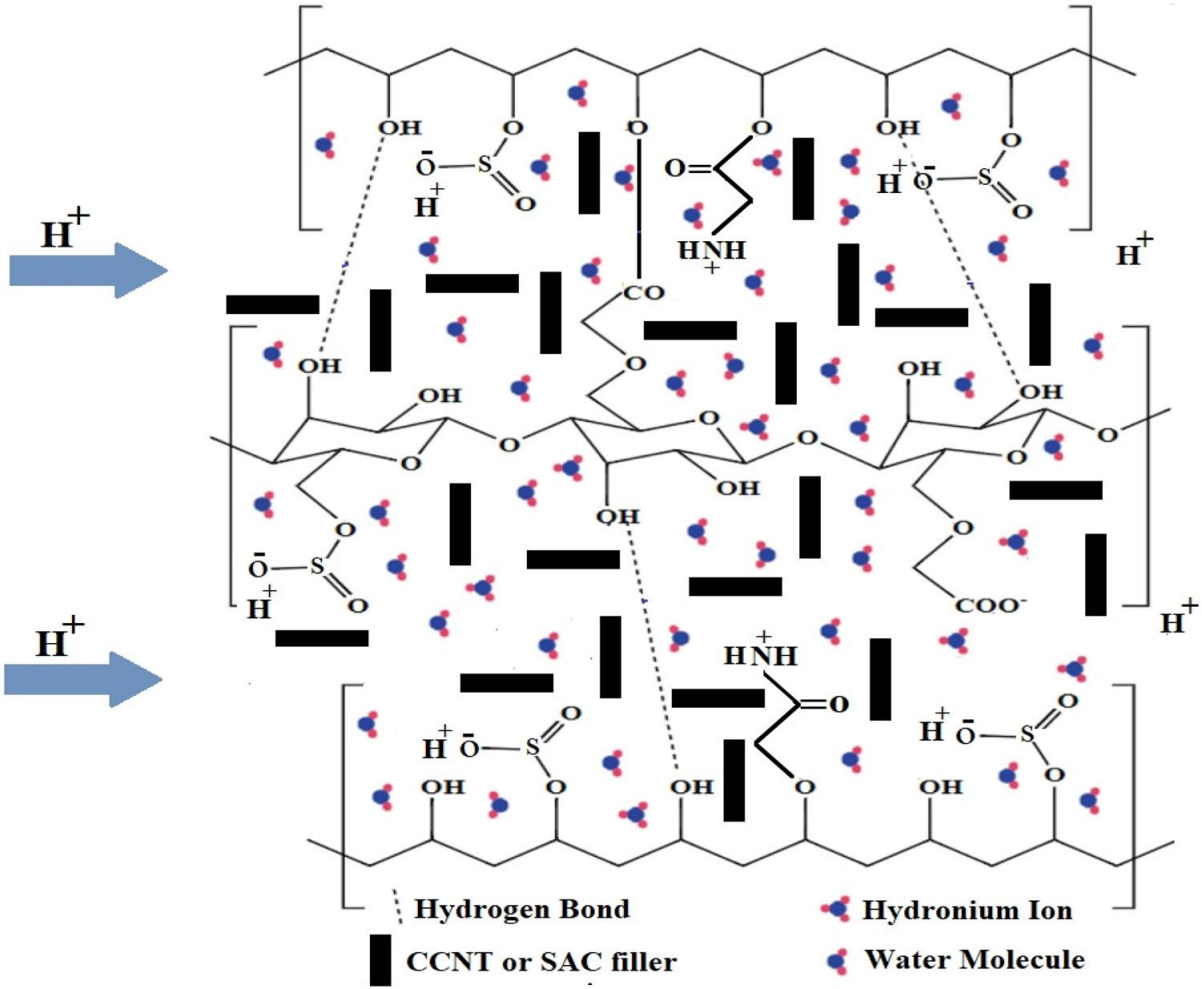
Illustrate the reaction route for CMC, PVA, and AA in addition (H^+^) hopping mechanism in the membrane.

Specified solutions of 10 wt% CMC, 10 wt% PVA, 10 wt% AA, and 2 wt% succinic acid solutions were prepared by dissolving the pre-weighed amount of each in de-ionized water at room temperature with stirring for 2, 8, 2 h, and 30 min, respectively till the solutions become homogenous. All solutions of CMC, PVA, AA, and succinic acid were stored at room temperature. The suggested reaction that takes place between membrane components was illustrated in (Scheme 1).

Sulfonated activated carbon (SAC) was prepared according to the method that was reported by Bermejo et al.^28^ with modification^29^ by mixing 10 g pure activated carbon with 100 mL of sulfuric acid, the mixture was stirred at 160 °C for 8 h. Then the mixture was washed several times with deionized water and allowed to dry at 60 °C for 6 h. Finally, SAC was packed and stored at room temperate for the next use.

The membrane was prepared by mixing 3 mL of CMC, 4 mL of PVA, 2 mL of AA, and 1 mL of succinic acid solutions then the mixture was vigorously stirred at 40 °C for 4 h. The previous steps were repeated to prepare 13 samples for use in the next steps. First, the various amounts (v/v %) of chlorosulfonic acid (CSA) (1, 2, 3, and 4%) were step-wise added to 4 samples during the stirring process, where the temperature was raised to 65 °C for 4 h. Second, 4% of CSA was added slowly to 4 samples under stirring conditions and temperature within 65 °C for 4 h. Then various amounts (w/v %) of CCNT (0.5, 1, 1.5, and 2%) were added under the previous condition for extra 2 h. Third, 4% of CSA was added slowly to 4 samples under stirring conditions and temperature within 65 °C for 4 h, then various amounts (w/v %) of SAC (0.5, 1, 1.5, and 2%) were added under the previous condition for extra 2 h. The remine sample is still without any additives. All mixtures were cast onto a polypropylene sheet. The solvent was removed by evaporation at room temperature for 12 h and then the casted membranes were allowed to dry at 70 °C for 6 h. Finally, the dried membranes were subjected to different characterization methods.

### Characterization

#### Spectral analysis

The chemical bonding and functional groups within the CMC/PVA/AA and modified CMC/PVA/AA based hybrid polyelectrolytic membranes were evaluated using (Shimadzu FTIR-8400 S, Japan) with a resolution of 4 cm^−1^ and a wavenumber range of 400–4000 cm^−1^^1^. Additionally, a laser Raman scattering spectrometer (SENTERRA-Bruker, Germany) equipped with a Leica microscope was also employed to investigate the chemical bonding and possible interactions inside the prepared polymeric membranes^30^.

#### Morphological features and microstructure

Morphological features and microstructure of CMC/PVA/AA and modified CMC/PVA/AA based hybrid polyelectrolytic membranes were investigated using scanning electron microscopic (SEM), (JEOL JSM-6360LA, Japan). SEM was in operation at an acceleration voltage of 15 kV. Magnification power varied from 500 to 5000^31,32^.

#### Mechanical properties

Tensile strength and the elongation at break of CMC/PVA/AA and modified CMC/PVA/AA based hybrid polyelectrolytic membranes have been conducted at ambient temperature using a universal testing machine (Shimadzu UTM, Japan) and the test was repeated three times for each membrane. Tensile and elongation measurements were carried out with cross-head movement at a constant speed of 3 mm/min for specimens of 30 × 10 mm^32^.

#### Methanol uptake

Methanol uptake of CMC/PVA/AA and modified CMC/PVA/AA based hybrid polyelectrolytic membranes are usually defined in weight percent with respect to the weight of the dried membrane. To determine the swelling ability specimens of these membranes were obtained by cutting specified membrane samples into 3 × 3 cm pieces, then the specimens were dried for 6 h at 90 °C, the dried sample weight is assigned as W_dry_. The dried samples were soaked in methanol for 12 h (equilibrium swelling) at room temperature to determine the methanol uptake ratio. Afterward, swelled samples were leached from methanol and weighted again (W_wet_). The swelling degree was determined by Eq. (1)^32,33^

1 $$Swelling\, degree \left(\%\right)=\frac{{w}_{wet}{ - w}_{dry}}{{w}_{dry}}\times 100$$

#### Gel fraction

The membranes were dried at room temperature to avoid any surface shrinking for 12 h and weighed (W_1_), then immersed in distilled water for another 24 h, up to an equilibrium swelling weight, to remove the leachable or soluble components. The polyelectrolytic membranes are then dried and weighed again (W_2_). The gel fraction (GF %) was carried out according to the method reported by Fahmy et al.^34^ and calculated by Eq. (2)

2 $$Gel\, Fraction \left(GF\%\right)=\frac{{w}_{2}}{{w}_{1}}\times 100$$

#### Ion exchange capacity

Ion exchange capacity (IEC) represents the total of active sites or functional groups responsible for ion exchange in CMC/PVA/AA and modified CMC/PVA/AA based hybrid polyelectrolytic membranes. In most cases, the IEC is determined using a standard acid–base titration technique. Weighed samples were immersed in 20 cm^3^ of a 2 M (NaCl) solution for 12 h at 25 °C. The solution was then titrated with a known concentration of NaOH. IEC (in mmol/g) is determined by using Eq. (3)^8,35^:

3 $$IEC (\mathrm{mmol}/g)=\frac{N(mmol/{cm}^{3}) \times V ({cm}^{3})}{W(gm)}$$

where N, V, and W are the concentration of the NaOH solution, the volume of the NaOH solution, and the weight of the sample, respectively.

#### Proton conductivity

The proton conductivity of the membranes was analyzed using impedance spectroscopy (Solartron 1260 gain phase analyzer, interfaced to a Solartron 1480 multistate). The test was conducted at a temperature of 60 °C. The Protonic conductivity (σ) of the membranes was calculated using Eq. (4) from impedance data^36^

4 $$\sigma (\mathrm{s}/\mathrm{cm})=\frac{L}{RWD}$$

where, (L) is the distance between electrodes, (R) is the membrane resistance, (D and W) are the thickness and width of the membranes, respectively.

#### Chemical stability analysis

The obtained CMC/PVA/AA-based membranes were investigated for oxidation stability through Fenton test^37^. The dried membranes specimen (1 × 4 cm^2^) was soaked in (2 ppm FeSO_4_ in 3% H_2_O_2_) for 1 h at ambient temperature. The weight in grams before and after immersing was determined as *W*_*o*_ and *W*_*f*_, respectively. The oxidation stability was calculated through Eq. (5):

5 $$Oxidation\, stability=\frac{{w}_{\circ }{ - w}_{f}}{{w}_{f}}\times 100$$

#### Fuel cell performance

The performance of the cell membrane was employed using an 890B commercial fuel cell system. The electrocatalysts Pt Ru/C (2 Pt: 1 Ru ratio) were coated into a different side of the CMC/PVA/AA-4%CSA-2%SAC membrane. The diffusion layer was assembled using two carbon paper sheets. The fuel cell unit was operated at 2 mL/min of 2 M methanol at the anode and 250 mL/min of oxygen at the cathode^37^.

## Results and discussion

### Investigation of the chemical composition of the membranes

#### FT-IR spectra

**Figure 1:**
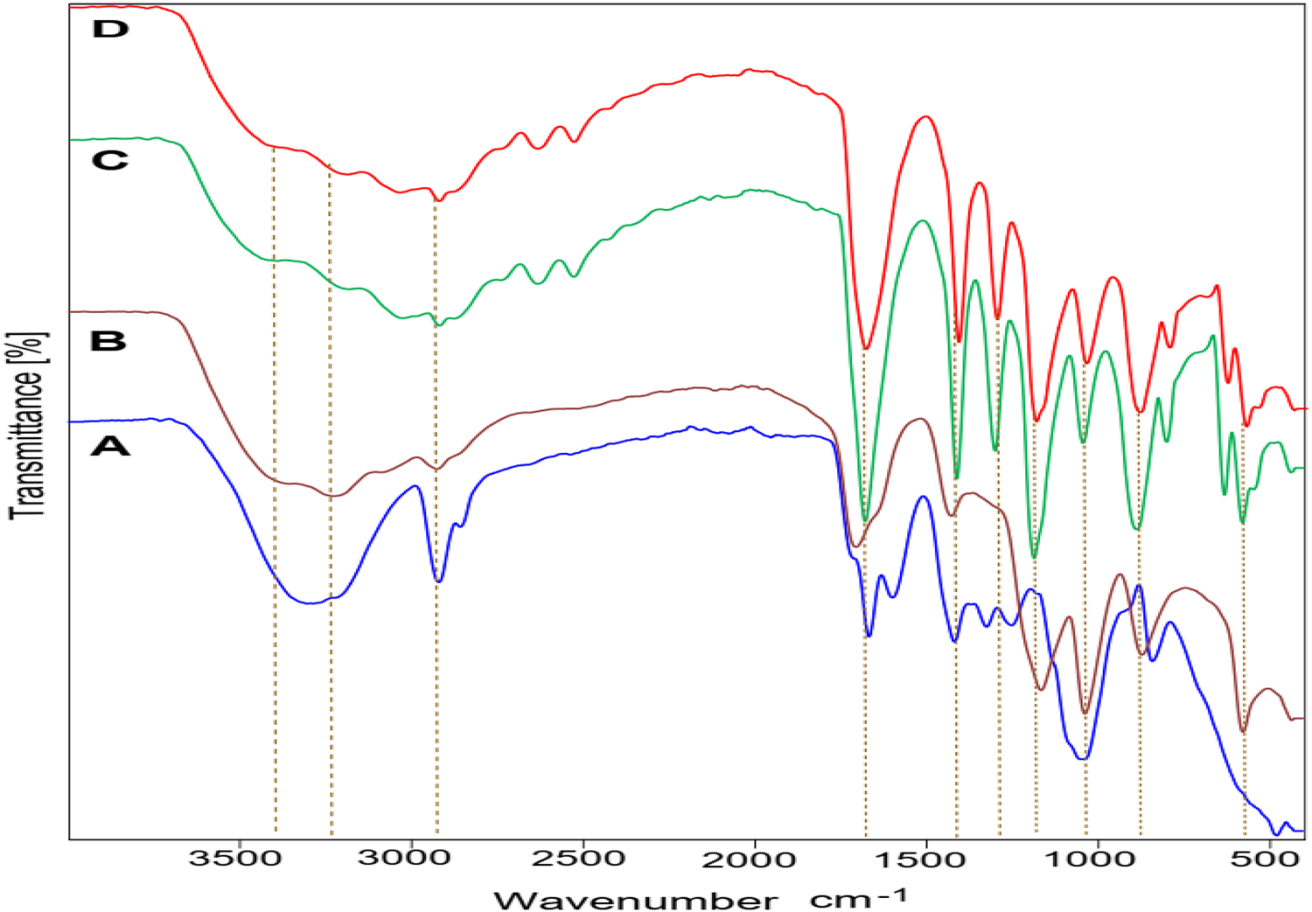
FT-IR spectra of CMC/PVA/AA based hybrid polyelectrolytic membrane (**A**) Virginal CMC/PVA/AA membrane. (**B**) CMC/PVA/AA-(4%) CSA. (**C**) CMC/PVA/AA -(4%) CSA-(2%) CCNT. (**D**) CMC/PVA/AA -(4%) CSA-(2%) SAC.

Figure 1 evidence FT-IR spectra of CMC/PVA/AA membrane and modified CMC/PVA/AA-based hybrid polyelectrolytic membrane using CSA, SAC and CCNT. The segments of IR peaks are reported in Table 1. The IR spectra of pristine CMC/PVA/AA membrane (Fig. 1-a) exhibit general peaks of –NH_2_, –OH, and C–H functional groups at 3411, 3302 and 2917 cm^−1^, respectively that found in polymeric blend backbone^38–40^.

Typical symmetrical stretching of –COO– bands of SU was observed at 1414 cm^−1^ (Fig. 1-a) which demonstrates the formation of crosslinked network and formation of ester covalent bond between CCNT and CMC/PVA polymeric blend (Fig. 1D)^41^. Moreover, the spectrum of –OH and –NH_2_ exhibits intensity decreases (Fig. 1B–D) which is assigned to the reaction of these groups with others during the sulfonation process or network formation^42^. The absorbance peaks at 1282, 1298 and 1297 cm^−1^ (Fig. 1B–D) resulting from CSA addition confirming the introduction of –SO_3_H groups to the CMC/PVA/AA polymeric matrix^43^.

Further confirmation, the stretching vibrational absorbed peaks at 620, 628 and 627 cm^−1^ also confirmed the presence of SO_3_^–2^ within the polymeric matrix^43^. Furthermore, the FT-IR spectrum of modified CMC/PVA/AA (Fig. 1B–D) possesses new bands at 572, 576 and 573 cm^−1^ which are assigned to carbonyl amide (–N–C=O) that confirm the covalent bond between nitrogen from AA and carboxylic group of CCNT and SU. These results verify the presence of –SO_3_H, –OH, –COO^−^, –NH, and –NCO^−^ groups within CMC/PVA/AA polymeric matrix^41,44^.

**Table 1:** The main FT-IR transmitted wavelength assignment of (A) virginal CMC/PVA/AA membrane.

Positions				Assignment
A	B	C	D	
3411	3443	3438	3436	–NH_2_ of acrylamide
3302	3224	3234	3239	O–H bending and H-bonding
2917	2922	2920	2921	C–H and symmetric CH_2_ stretching of the polymeric backbone
1663	1701	1677	1680	C–O and C=O of CCNT
1414	1423	1410	1410	COO– of SU and C–N of AA
–	1282	1298	1297	O=S=O^45^
	1161	1184	1183	C–C bond
1034	1033	1041	1037	C–O of pyranose ring of CMC
836	867	885	882	O–H bending
–	–	797	796	C–H deformation
–	620	628	627	SO_3_^–2^ group
–	572	576	573	N–C=O bond

(B) CMC/PVA/AA-(4%) CSA. (C) CMC/PVA/AA-(4%) CSA-(2%) CCNT. (D) CMC/PVA/AA-(4%) CSA-(2%) SAC.

#### Raman spectroscopy

**Figure 2:**
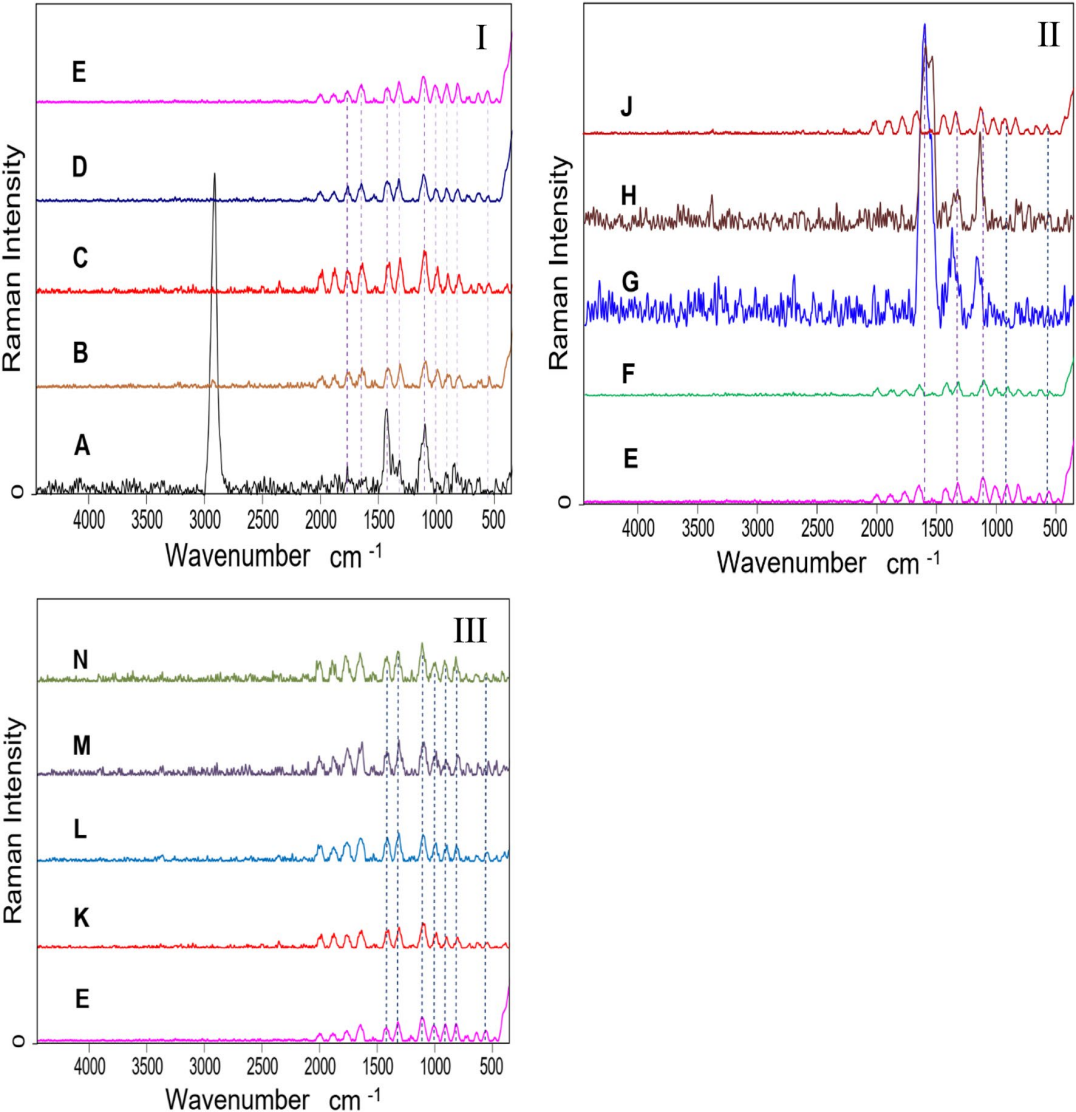
(**I**) Raman spectra of (**A**) CMC/PVA/AA, (**B**) CMC/PVA/AA-(1%) CSA, (**C**) CMC/PVA/AA-(2%) CSA, (**D**) CMC/PVA/AA-(3%) CSA, (**E**) CMC/PVA/AA-(4%) CSA. (**II**) Raman spectra of (**E**) CMC/PVA/AA-(4%) CSA, (**F**) CMC/PVA/AA-(4%) CSA-(0.5%) CCNT. (**G**) CMC/PVA/AA-(4%) CSA-(1%) CCNT. (**H**) CMC/PVA/AA-(4%) CSA-(1.5%) CCNT. (**J**) CMC/PVA/AA-(4%) CSA-(2%) CCNT. (**III**) Raman spectra of (**E**) CMC/PVA/AA-(4%) CSA. (**K**) CMC/PVA/AA-(4%) CSA-(0.5%) SAC. (**L**) CMC/PVA/AA-(4%) CSA-(1%) SAC. (**M**) CMC/PVA/AA-(4%) CSA-(1.5%) SAC. (**N**) CMC/PVA/AA-(4%) CSA-(2%) SAC.

To find out more about the membrane microstructure, the nondestructive Raman spectroscopy technique was used as shown in Fig. 2. The absorbed peak at 605 cm^−1^ that appeared in all Raman curves except the curve (A) is related to the C–S covalent bond and this band increases in intensity (Fig. 2I-A) with the increase of CSA molar ratio resulting in increases of –SO_3_H groups^46^. The other strong vibrational peak that appeared around 900 cm^−1^ was attributed to the C–C of the polymeric chain. Other signals that have emerged at 1099 cm^−1^ due to the asymmetric vibration of C–O–C bonds confirm the cross-linking reactions^47^. In addition, a strong valance band at 1634 cm^−1^ owing to the C=O group and the intensity of this band decreases with the functionalization process of CMCC/PVA/AA with CSA, CCNT and SAC^48^.

It is worth recalling that all spectra of Fig. 2II,III are quite similar with the same pattern, as a result of the structure of CCNT and SAC does not undergo major damage during the preparation and modification process^49^.

### Topography of the membranes

**Figure 3:**
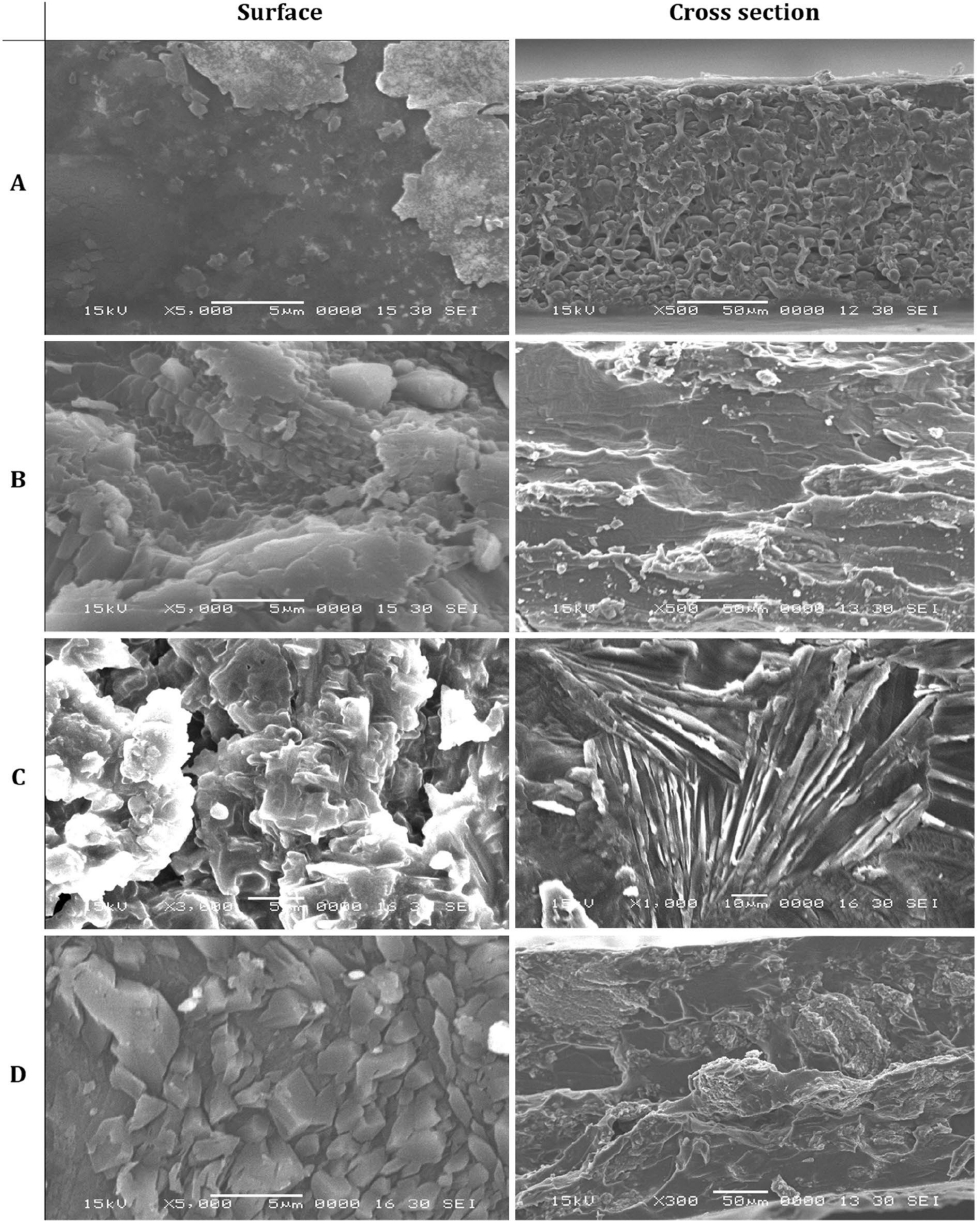
SEM micrographs of surface and cross-section of CMC/PVA/AA and modified CMC/PVA/AA based hybrid polyelectrolytic membranes. (**A**) pristine CMC/PVA/AA membrane. (**B**) CMC/PVA/AA-(4%) CSA. (**C**) CMC/PVA/AA-(4%) CSA-(2%) CCNT. (**D**) CMC/PVA/AA-(4%) CSA-(2%) SAC.

Surface and cross-section SEM images of the membranes were presented in Fig. 3. As a result membrane has smooth and glossy-like surface morphology and good blend compatibility without any phase separation or cracking passed across the membrane as a result of using SU as a cross-linker agreeing well with previous works^50^. A part of the membrane exhibits an erratic surface as a result of some side reaction between the CMC/PVA/AA polymeric blend. As shown in Fig. 3B, no observed defects in the membrane surface and cross-section during the sulfonation process. The membrane possesses some irregularity as a result of sliding of polymeric chains and large CMC/PVA/AA matrix deformation during the production process^49^ and strong intramolecular bonding between polymeric ionic groups as –SO_3_H, OH, and NH^50^. This irregularity also extended to CMC/PVA/AA-(4.0%)-(2.0%) CCNT (Fig. 3C) and CMC/PVA/AA-(4.0%)-(2.0%) SAC (Fig. 3D) membranes. This is an advantage property that can protect CCNT and SAC structures from great damages that can decrease their reinforcement action in the membrane^49^.

Consequently, this gained property improves the protonic conductivity of membranes through forming of micro-channels around CCNT and SAC blends for water and hydrogen ions^13^. It is worth mentioning that the membranes exhibit good CCNT and SAC dispersibility as a result of the pre-functionalization of the blend before being used in a membrane preparation. Some of the changes in membrane surface and gathered of the blend are due to the creation amendment in matrix molecular arrangement after CCNT and SAC addition that also improves crosslinking behaviour^49^.

### Elemental analysis

The elemental composition of CMC/PVA/AA and modified CMC/PVA/AA based hybrid polyelectrolytic membranes were determined through a Carbon, hydrogen, nitrogen, and sulfur (CHNS) analyzer and the obtained results were presented in (Table 2. With the different concentrations of CSA in CMC/PVA/AA (Table 2I), the sulfur content is increased with increasing the molar ratio of CAS in the membrane suggesting an increase of –SO_3_H groups attached to the polymeric backbone. Adversely, carbon and hydrogen content exhibit slight loss with the CSA addition. The obtained behaviour confirmed the modification of CMC/PVA/AA by sulfonation which takes place without any undesired degradation or fragmentation which is also assured by the C/N and C/H ratio^51,52^. The amount of carbon is increased with increasing the amount of CCNT in CMC/PVA/AA membrane and the hydrogen percent shows a slight change (Table 2II). This can be explained through the reaction of –COOH group of CCNT with –OH group of PVA to form an ester bond through the elimination of H_2_O molecule causing the decrease of hydrogen ratios.

**Table 2:** Elemental composition of CMC/PVA/AA-based hybrid polyelectrolytic membranes with different concentrations of CSA, CCNT and SAC.

	Sample	Elemental Content %					
		N	C	H	S	C/N	C/H
I	0% CSA	2.2	43.46	7.005	0.104	19.712	6.204
	1% CSA	2.82	38.50	6.394	3.025	13.646	6.021
	2% CSA	2.84	33.19	6.258	5.816	11.696	5.303
	3% CSA	2.38	29.94	5.936	7.577	12.576	5.044
	4% CSA	1.89	25.02	6.192	9.010	13.216	4.041
II	4% CSA-0.5% CCNT	2.32	26.29	5.464	8.715	11.331	4.811
	4% CSA-1% CCNT	2.27	29.05	5.752	8.717	12.798	5.050
	4% CSA-1.5% CCNT	2.24	25.36	5.629	9.587	11.321	4.506
	4% CSA-2.0% CCNT	2.09	31.43	5.532	7.685	15.065	5.682
III	4% CSA-0.5% SAC	2.61	26.16	5.441	9.307	10.022	4.807
	4% CSA-1.0% SAC	2.7	27.86	5.648	9.61	10.318	4.9328
	4% CSA-1.5% SAC	2.54	28.1	5.704	9.682	11.063	4.926
	4% CSA-2.0% SAC	2.31	29.53	5.456	9.944	12.688	5.412

The amount of sulfur (that related to –SO_3_H groups) content was not greatly affected during the addition of CCNT indicating good stability of –SO_3_H groups. The influence of SAC addition to CMC/PVA/AA-(4%) CSA has been presented in (Table 2III). It was noted that an increase in the amount of carbon and sulfur percent with the addition of SAC resulting more –SO_3_H groups attached to the membrane that will improve the ionic conductivity. Therefore, the slight variations in C/H and C/N% ratios give a positive snapshot of no major change in the membrane structure and agree well with previous work^53^.

### Mechanical properties

It is important to study the mechanical performance of the membranes to identify the ability of membranes to withstand operating conditions of DMFC or another kind of FC and resist rapid breakout. Tensile strength and ultimate elongation give an impression of the extent of mechanical for membranes. The tensile strength and elongation results were summarized in Table 3.

**Table 3:** Tensile strength and elongation at break of CMC/PVA/AA and modified CMC/PVA/AA hybrid polyelectrolytic membranes with different amounts of CCNT and SAC.

Membrane	Tensile strength (MPa)	Elongation at break (%)
CMC/PVA/AA	23.41 ± 0.82	283.22 ± 6
CMC/PVA/AA-1%CSA	42.81 ± 1.61	394.50 ± 10
CMC/PVA/AA-2%CSA	41.52 ± 1.46	416.85 ± 22
CMC/PVA/AA-3%CSA	37.20 ± 1.74	441.32 ± 16
CMC/PVA/AA-4%CSA	31.11 ± 2.03	463.07 ± 17
CMC/PVA/AA-4%CSA-0.5% CCNT	33.50 ± 0.75	432.40 ± 23
CMC/PVA/AA-4%CSA-1.0% CCNT	32.54 ± 1.33	394.33 ± 11
CMC/PVA/AA-4%CSA-1.5% CCNT	26.73 ± 0.45	381.61 ± 6
CMC/PVA/AA-4%CSA-2.0% CCNT	24.81 ± 1.01	312.40 ± 9
CMC/PVA/AA-4%CSA-0.5% SAC	37.22 ± 1.18	443.13 ± 14
CMC/PVA/AA-4%CSA-1.0% SAC	36.04 ± 1.22	422.50 ± 14
CMC/PVA/AA-4%CSA-1.5% SAC	32.22 ± 0.92	374.23 ± 12
CMC/PVA/AA-4%CSA-2.0% SAC	30.71 ± 0.57	331.86 ± 8

As shown in (Table 3), the tensile strength of pristine CMC/PVA/AA of 23.41 MPa which is the lowest value and might be related to the low interactions between CMC, PVA, and AA polymeric components that also leads to low stretching and elongation at break. In presence of 1.0% CSA, the tensile and elongation values are 42.81 and 394.5, respectively. This behaviour is attributed to the insertion of more ionic -SO_3_H groups that increase the intramolecular forces between polymeric components^54^ rather than covalent interactions. With rising the molar ratio of CSA in the membrane to 4% CSA, the tensile shows a mild decrease to 32.11 MPa which can be illustrated by the deterioration impact of CSA addition especially at a higher molar ratio^55^. The ultimate elongation shows inverse action by increasing the 394.5–463.07% resulting in more polymeric segment mobility^56^.

With increasing the molar ratio of CCNT and SAC in CMC/PVA/AA-(4%) CSA the tensile strength displays mild decline. It can be clarified according to that the mechanical properties of prepared membranes are affected by molecular arrangement^57^ which is further influenced by the introducing of CCNT and SAC filler^58^. The addition of fillers leads to reduction in crystallinity leading to reduce the tensile strength values. In addition, the low adhesion between polymeric matrix and filler^59^ and more polymeric segmental restricting especially at CCNT addition because it has a low degree of carboxylation compared to SAC has more –SO_3_H groups might be the explanation. So, the SAC-based membrane exhibits higher strength than CCNT-based membranes. In general, the mechanical strength findings of modified CMC/PVA/AA hybrid polyelectrolytic membranes had proper stamina for DMFC and withstand operating conditions^60^.

### Methanol uptake

**Figure 4:**
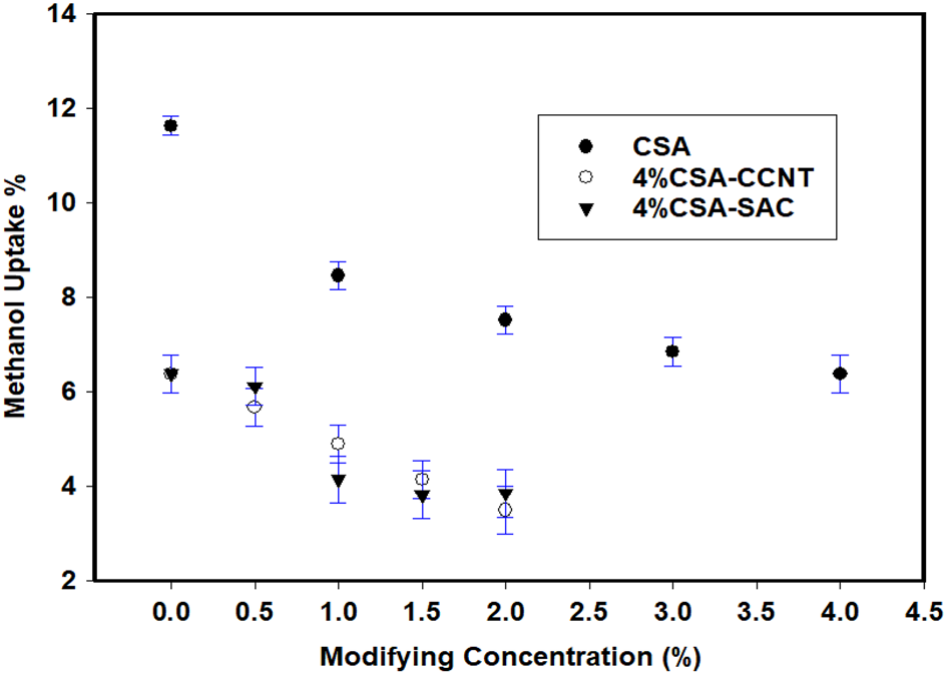
Methanol uptake of CMC/PVA/AA and modified CMC/PVA/AA hybrid polyelectrolytic membranes with different amounts of CSA, CCNT and SAC.

The capacity of a polymer membrane for methanol uptake strongly depends on its affinity and gaps present within the structure of the membrane available for uptake of liquids^61^. Figure 4 displays the amount of methanol taken by CMC/PVA/AA and modified CMC/PVA/AA based hybrid polyelectrolytic membranes as a function of different molar ratios of CSA, CCNT and SAC. As shown, the pristine CMC/PVA/AA membrane possesses higher methanol uptake (11.6%) due to the high affinity of the membrane to methanol as a result of the high methanophilic -OH and -COOH groups. Upon sulfonation, the amount of methanol taken by the membrane decreased with rising the molar ratio of CSA reached 6.4% at 4% CSA due to the decrease of the membrane affinity to methanol as a result of introducing of low methanophilic sulfonic –SO_3_H groups.

Upon incorporation of CCNT and SAC blend to CMC/PVA/AA membrane the amount of methanol taken possesses lower values of 5.7 and 6.1%, respectively as result of the blocking effect that occurred by CCNT and SAC filler. As the amount of CCNT and SAC grew to 2% w/v the amount of taken methanol progressively decreased and reached 3.49 and 3.85%, respectively. The ability of CCNT and SAC to block methanol channels and decreases membrane porosity offering low available space within CMC/PVA/AA membranes might be the reason and agree well with data obtained for Nafion/SPA^62^. It is worth noting, Yao et al*.*^37^ reported that introducing sulfonated nanosheets into polymeric membrane matrix not only improves the ionic conductivity but also inhibits the penetration of methanol resulting in microstructure distribution.

### Gel fraction

**Figure 5:**
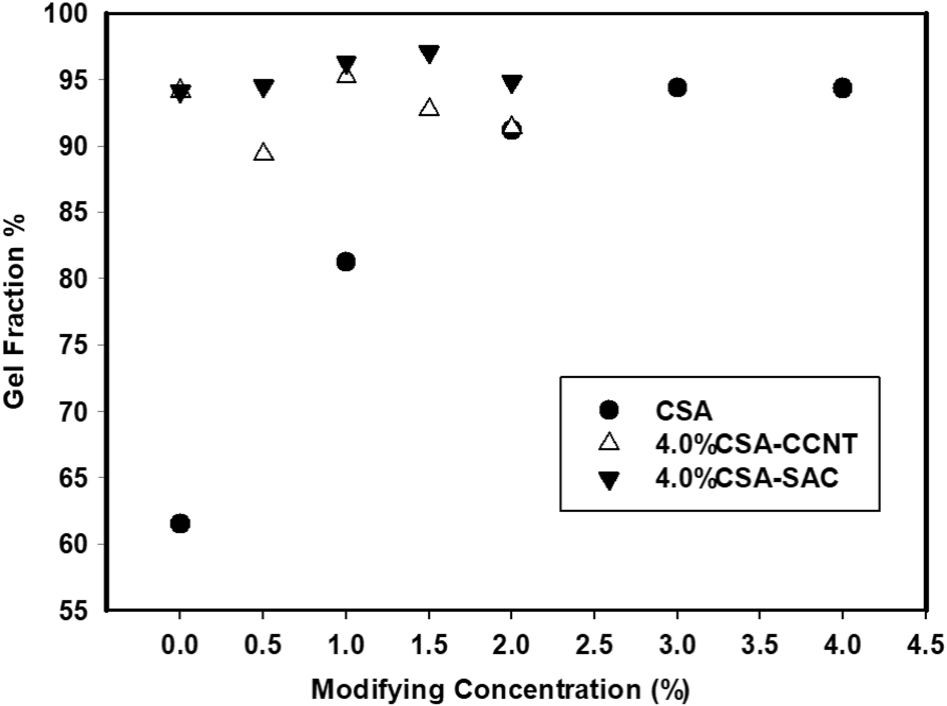
Gel fraction of CMC/PVA/AA and modified CMC/PVA/AA hybrid polyelectrolytic membranes with different amounts of CSA, CCNT and SAC.

Gel fraction analysis of CMC/PVA/AA based hybrid polyelectrolytic membranes was carried out to figure out the uncross-linked membrane components and provides an impression about crosslinking density^63^. It was observed that the gel fraction was strongly affected by modifications of membranes as shown in Fig. 5. Gel fraction ratio exhibits a lower ratio of 61.5% for the pristine CMC/PVA/AA membranes. With raising the amount of CSA in CMC/PVA/AA membrane from 1 to 4% v/v, the gel fraction percent was changed from 88.3 to 94.3% due to the increasing crosslinking density as a result of the sulfonation process that allowed for more –SO_3_H group attached to the membrane. Further modification process using CCNT and SAC the amount of gel fraction increased to 91.4% and 94.9%, respectively with a maximum loading due to the crosslinking improvement^64^.

### Ion exchange capacity (IEC)

**Figure 6:**
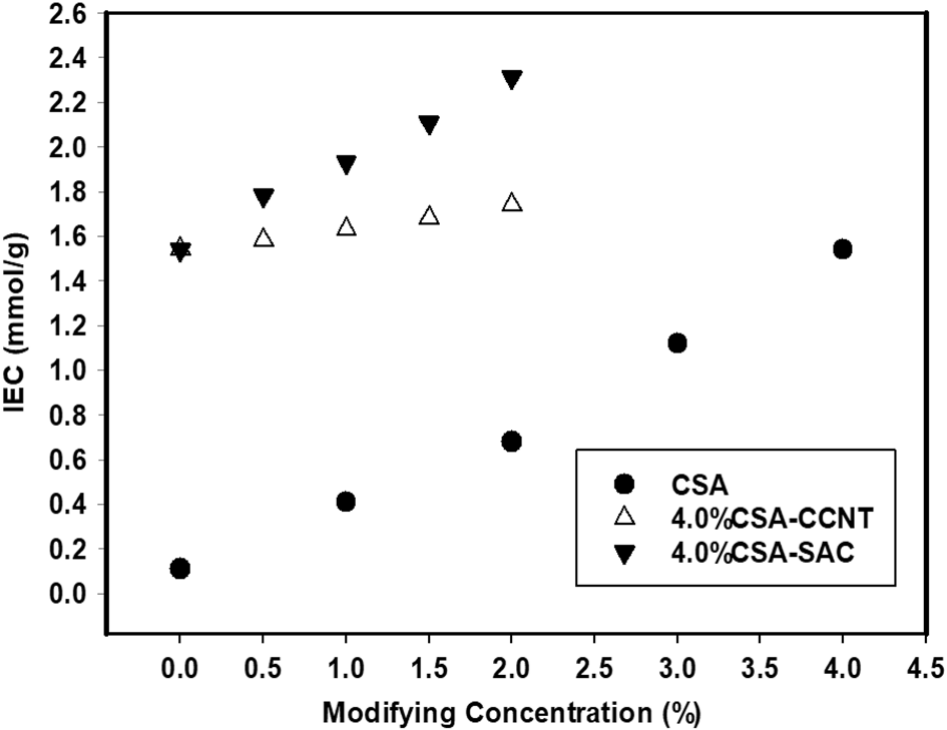
IEC of CMC/PVA/AA and modified CMC/PVA/AA hybrid polyelectrolytic membranes with different amounts of CSA, CCNT and SAC.

IEC is a conventional method employed as an indicator for membrane ionic group content those responsible for holding and transporting protons from anode to cathode^65^. The IEC value increased dramatically with increasing the amount of CSA in CMC/PVA/AA membranes reaching 1.54 mmol/g at 4% (v/v) CSA. It can be explained as the increasing of –SO_3_H ionic groups on CMC/PVA/AA based membranes leading to high reactive and exchangeable sites (Fig. 6). Additionally, the modification of CMC/PVA/AA based membranes using CCNT and SAC effects on the IEC values by increasing. When the amount of CCNT and SAC increased in those sulfonated membranes from 0 to 2% (w/v) IEC changed to 1.74 and 2.31 mmol/g respectively as a result of increasing the interference area. In SAC-based membranes the IEC values aren’t higher than other CCNT-based membranes only but higher also than Nafion 115 (0.91 mmol/g) due to the concentration of sulfonic groups^66^.

### Proton conductivity (PC)

**Figure 7:**
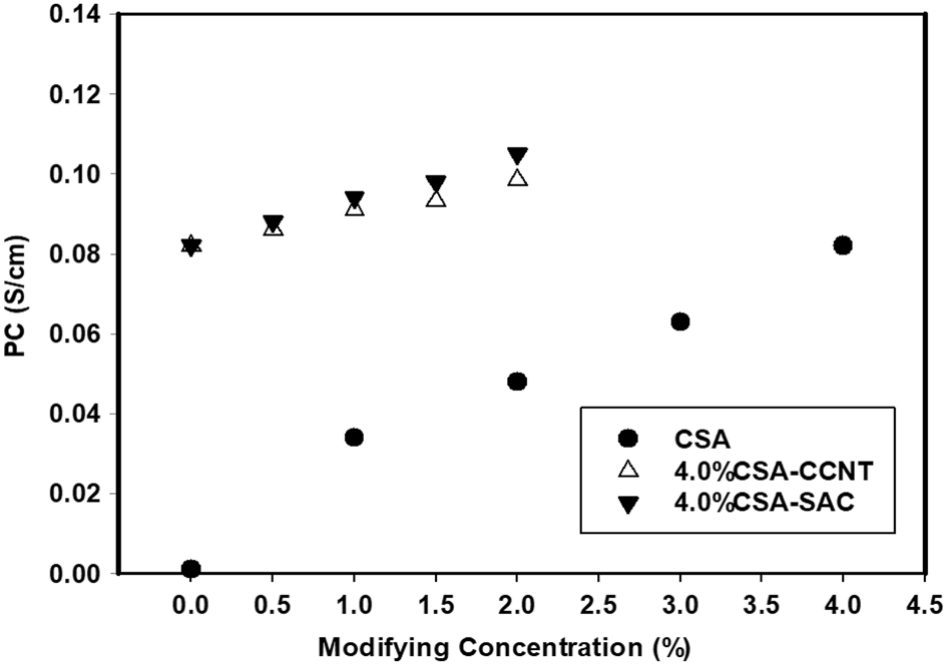
PC of CMC/PVA/AA and modified CMC/PVA/AA hybrid polyelectrolytic membranes with different amounts of CSA, CCNT and SAC at 60 °C.

PC is one of the most important investigations of FC membranes which decided their effectiveness. For the pristine membrane, the PC value was 0.001 S/cm due to the ionic groups found in lower ratio (Fig. 7).

In the case of CSA, addition the number of sulfonic groups is increased with raising the molar ratio of CSA in the membrane reached to 0.082 S/cm with a maximum loading of CSA 4% (v/v). The presence of –SO_3_^−^H^+^ groups as a result of CSA addition which act as the protonic carrier is the key^67^. Similarly, the addition of CCNT and SAC fillers to sulfonated CMC/PVA/AA membranes leads to the increase of PC to 0.098 and 0.105 S/cm, respectively owing to the presence of those fillers providing an ionic channel pass way and a large surface area especially at functionalized SAC. This funding is inherent to that obtained by IEC^26^.

### Chemical stability analysis

**Figure 8:**
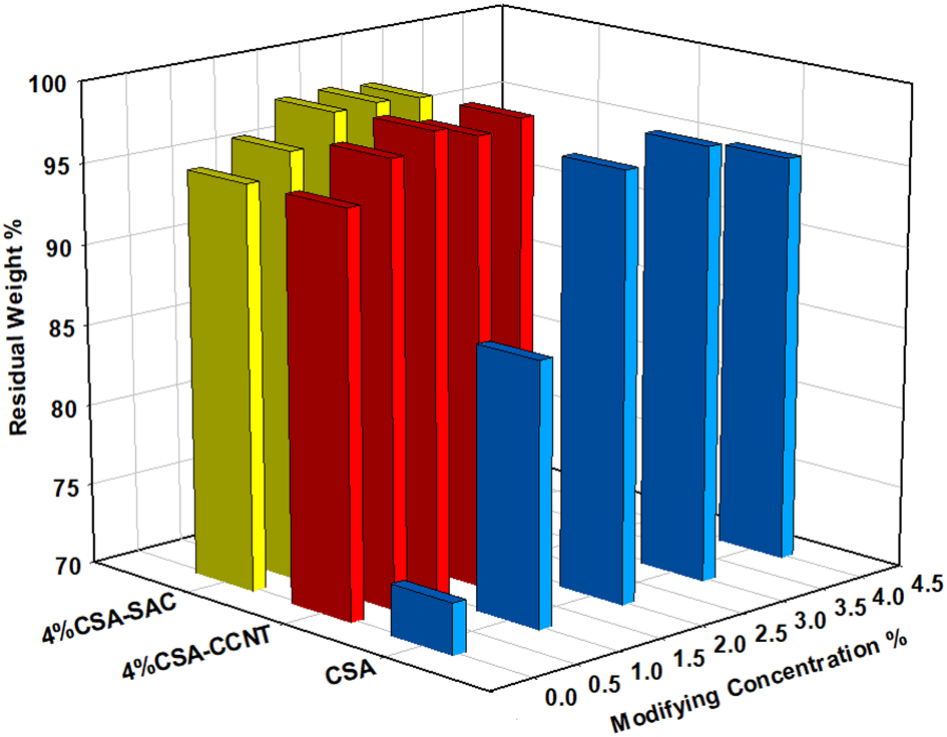
Oxidation stability of CMC/PVA/AA and modified CMC/PVA/AA hybrid polyelectrolytic membranes with different amounts of CSA, CCNT and SAC.

The polymer electrolyte membrane durability is highly dependent on the resistance to oxidation. The stability of CMC/PVA/AA-based membranes in Fenton’s reagent to oxidation was investigated and the obtained results were illustrated in Fig. 8. Generally, all membranes expose oxidation stability with increasing the amount of modifying agent CSA, CCNT and SAC. This behaviour is attributed to the presence of oxygen enrichment groups such as –SO_3_H, –OH, and –COOH with in polymeric matrix. These oxygen groups are leading to an increase of the membrane resistance to oxidation through the improvement of cross-linking density by the formation of hydrogen bonds which hinder the free radical attacks^37,68,69^. In addition, the presence of CCNT and SAC increase the surface area that leads to improve the oxidative stability as that reported by Yao et al.^37^.

#### Thermal stability analysis

**Figure 9:**
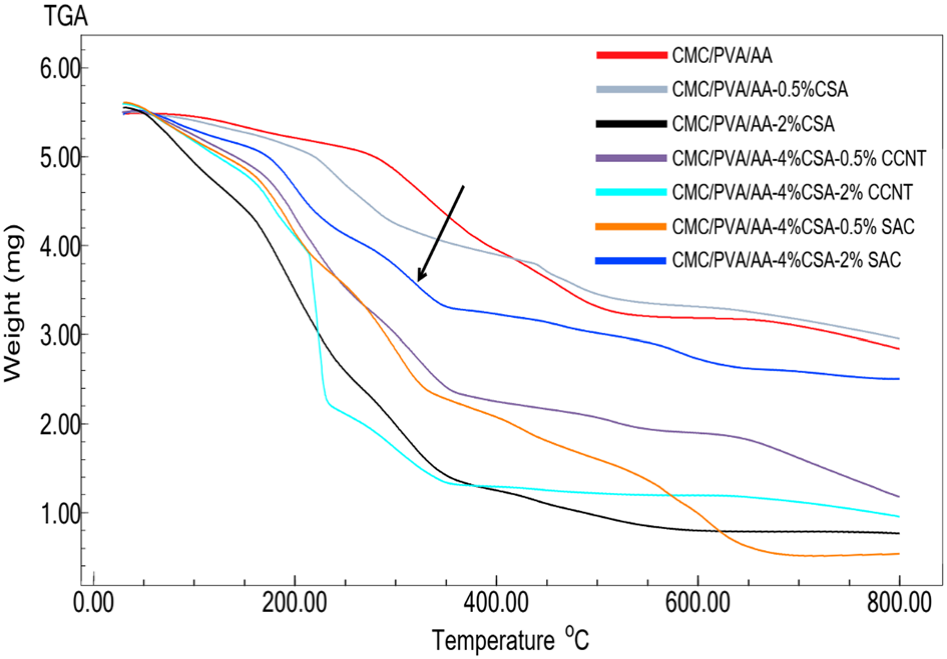
Thermogravimetric spectra of CMC/PVA/AA and modified CMC/PVA/AA hybrid polyelectrolytic membranes with different amounts of CSA, CCNT and SAC.

The thermal stability of obtained CMC/PVA/AA-based hybrid polyelectrolytic membrane was estimated through (204 Phoenix TGA instrument (NETZSCH, Germany) at heating rate of 10 °C/min under N_2_ atmospheric. As shown in Fig. 9, the thermograms reveal three regions of weight loss. The first region has taken place from 60 to 200 °C in which the physically bonded water and solvent are volatile at the beginning of this region. Evaporation of chemically bonded water and degradation of –NH_2_ groups occurred at the end of the first period^70^. The second region of weight loss (200–350 °C) is associated with the destruction of side polymer groups such as sulfonic groups and other pendant groups. The main polymer chains possess destruction with increasing temperature. The breakage of C–C bonds usually started at a temperature of 350 °C and is occupied with C–H, O–H and C=O bonds at elevated temperatures^70^. The smooth transition between sequential regions indicates that the ion-conducting groups are able to remain long time bonded to the main polymer matrix when exposed to different temperature range^68^.

In general, the incorporation of CCNT and SAC had not clearly affected on the thermal stability as a result of low filler content and good blend compatibility. It is worth mentioning that the addition of SAC filler in the higher ratio (2% wt) leads to an improvement of the thermal stability compared to CCNT filler.

#### Membrane cell performance

**Figure 10:**
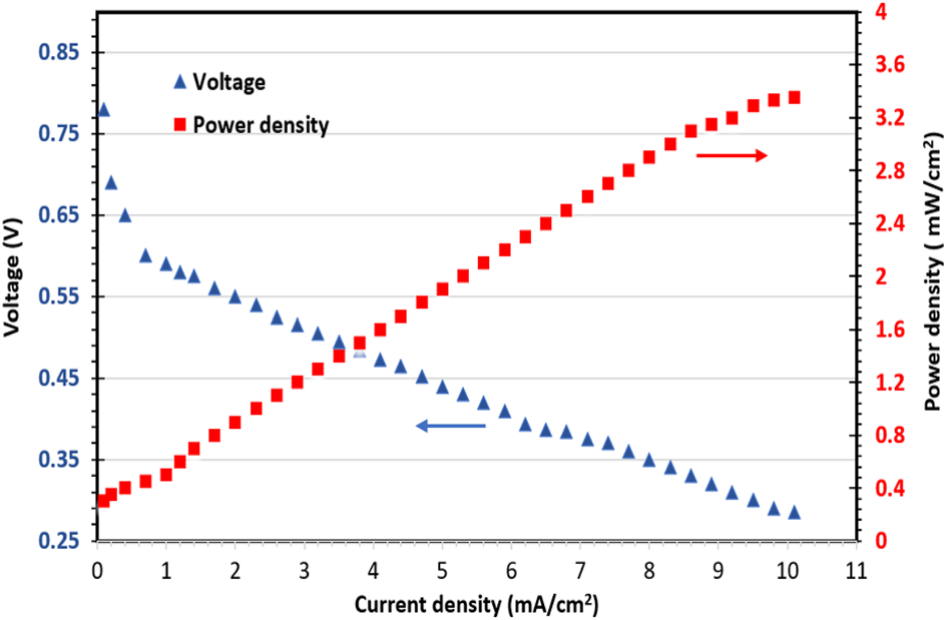
Polarization curves and power density of DMFC with CMC/PVA/AA-4%SAC-2%SAC hybrid polyelectrolytic membrane.

High ion exchange capacitor and conductor combined with good mechanical stability and low methanol uptake of the modified CMC/PVA/AA-based membranes makes these membranes suitable as polymeric electrolyte membrane for DMFC^37^. The performance of cell was performed at 60 °C with 2 M methanol at the anode. CMC/PVA/AA-4%CSA-2%SAC is selected to be membrane electrode assemblies and the obtained results for voltage and power density are presented in Fig. 10. The cell possesses maximum power density and voltage (3.35 Mw/cm^−2^ and 0.78 V, respectively). These findings demonstrate that the modification process of CMC/PVA/AA-based membranes can be improved the cell performance.

## Conclusion

A hybrid polyelectrolytic membrane for DMFC made from a CMC/PVA/AA based polymeric blend and modified with different concentrations of CSA, CCNT, and/or SAC with the addition of succinic acid as a crosslinker. The hybrid polyelectrolytic membrane was successfully prepared through solution casting techniques. The structural and functional properties of the prepared hybrid membrane were proven by FT-IR, Raman spectra, and CHNS analysis. Other physicochemical properties were determined by measuring methanol uptake, gel fraction, tensile strength, elongation, ion exchange capacity, proton conductivity, thermal and chemical stability. the characterization results evidenced that the modification process using CSA, CCNT, and SAC has achieved the purpose of which used to induced –SO_3_H groups that converted CMC/PVA/AA from proton poor conductor into a good proton conductor and capacitor as well as good mechanical and oxidation stability to withstand the operating conditions. The IEC values are increased with increasing sulfonation levels of CMC/PVA /AA and the proportion of CCNT or SAC in the polymer blend. IEC (2.31 mmol/g), protonic conductivity (0.105 S/cm) and cell performance (power density 3.35 Mw/cm^−2^ and voltage 0.78 V) reached their maximum in the case of CMC/PVA/AA-(4%) CSA-(2%) SAC blend-based membrane. In addition, the incorporation of CCNT and SAC fillers significantly improved the membrane properties as mechanical stability and lowered methanol uptake made the membrane have good barrier properties to methanol cross-over. Eventually, the CMC/PVA/AA based membranes possess acceptable electrochemical performance e.g., IEC, protonic conductivity, thermal, mechanical, and chemical stability making them crucial for further low-cost DMFC applications.

## Data Availability

All data generated or analysed during this study are included in this published article.
